# Sunflower (*Helianthus annuus* L.) Plants at Various Growth Stages Subjected to Extraction—Comparison of the Antioxidant Activity and Phenolic Profile

**DOI:** 10.3390/antiox9060535

**Published:** 2020-06-19

**Authors:** Francesco Gai, Magdalena Karamać, Michał A. Janiak, Ryszard Amarowicz, Pier Giorgio Peiretti

**Affiliations:** 1 Institute of Sciences of Food Production, National Research Council, 10095 Grugliasco, Italy; francesco.gai@ispa.cnr.it (F.G.); piergiorgio.peiretti@ispa.cnr.it (P.G.P.); 2 Institute of Animal Reproduction and Food Research, Polish Academy of Sciences, Tuwima 10, 10-748 Olsztyn, Poland; m.janiak@pan.olsztyn.pl (M.A.J.); r.amarowicz@pan.olsztyn.pl (R.A.)

**Keywords:** aerial parts, morphological stages, scavenging activity, reducing power, emulsion oxidation, chlorogenic acid, dicaffeoylquinic acid

## Abstract

The aim of this study was to evaluate the differences in the antioxidant activity and phenolic profile of sunflower (*Helianthus annuus* L.) extracts obtained from the aerial parts of plants harvested at five growth stages. In vitro assays were used to determine the antioxidant activity, i.e., ABTS^•+^ and DPPH^•^ scavenging activity, the ferric-reducing antioxidant power (FRAP) and the ability to inhibit β-carotene–linoleic acid emulsion oxidation. Phenolic compounds, such as mono- and dicaffeoylquinic acid isomers and caffeic acid hexose, were identified using the LC–TOF–MS/MS technique. The predominant compound during the growth cycle of the plant was 3,5-di-*O*-caffeoylquinic acid, whose content was the highest at the mid-flowering stage. The total phenolic content was also the highest in sunflowers at the mid-flowering stage. The main phenolic compound contents were closely correlated with ABTS^•+^ and DPPH^•^ scavenging activity and FRAP. No significant correlation was found between the total phenolic content and the antioxidant activity in the emulsion system. The highest antiradical activity and FRAP were generally determined in older plants (mid-flowering and late flowering stages). In conclusion, the aerial parts of sunflowers, in particular those harvested at the mid-flowering stage, are a good plant material from which to obtain phenolic compound extracts, albeit mainly of one class (esters of caffeic acid and quinic acid), with high antioxidant activity.

## 1. Introduction

There is scientific evidence that the overproduction of reactive oxygen species (ROS) in cells of the body beyond those needed for the effectiveness of the antioxidant defense system may cause damage to such biomolecules as lipids, proteins and DNA, and as a consequence may lead to various degenerative diseases, including cancer, diabetes mellitus, cardiovascular disease, hypertension, rheumatoid diseases, arthritis and neurodegenerative diseases [1,2,3]. The consumption of antioxidants in food and dietary supplements has been linked to a reduced risk of these diseases [4,5]. Antioxidants also play an important role in extending the shelf life of food [6,7,8]. Utilized as additives, they limit the oxidation of food product ingredients, especially lipids. The increasing interest in new sources of natural antioxidants is thus justified, considering the above and general trend of using natural substances to replace synthetic ones.

Sunflower (*Helianthus annuus* L.) is a short season plant that is native to North America and is currently grown worldwide. It is generally planted for seed and oil production purposes. Sunflower seeds are the fourth largest source of edible oil after soybean, rapeseed and peanut [9]. In order to obtain good quality seeds, sunflowers should be harvested after reaching physiological maturity with a moisture content of about 10–13% [10]. However, younger plants can also constitute valuable agricultural material. Green sunflower plants are used as forage and a silage source by livestock producers because of their nutritional quality, that is, high protein and fat contents [11,12,13]. Interestingly, young sunflower shoots and florets have long been used in traditional medicine to prepare teas and tinctures, which generally have anti-inflammatory effects [14].

The antioxidant potential of sunflower seed kernels and hulls, as well as of the seed oil pressing by-product (cakes), has been recognized [15,16,17]. This potential has been found to be high compared to that reported for other common oilseeds and nuts [18]. Phenolic compounds are mainly responsible for the antioxidant potential of sunflower seeds [19,20]. Among these compounds, chlorogenic acid, other caffeoylquinic acid isomers and their derivatives and caffeic acid and its derivatives, together with *p*-coumaroyl and feruloylquinates, and more rarely, flavonoids, have been identified [19,20,21,22]. Less knowledge is available about the bioactivity of the phytochemicals of other parts of sunflowers. Liang et al. [23] determined the composition of phenolic compounds of ray and disk florets and found that the main constituents were hydroxycinnamic acid derivatives, with 1,5-dicaffeoyquinic acid being predominant. The same group of researchers reported that these compounds were a major contributor to the antioxidant activity of both floret extracts [24]. Recent information about the secondary metabolites of sunflower leaves has been provided as a result of metabolomic studies, in which compounds from three chemical groups, including hydroxycinnamoylquinates, methyl-flavonoids and sesquiterpenoids, were detected [25,26]. Onoja et al. [27] found that a sesquiterpene lactone, isolated from *H. annuus* leaves, had antidiabetic and antioxidant properties, but, to the best of our knowledge, the total antioxidant potential of sunflower leaves has not yet been estimated.

The presence of phenolic compounds in the main morphological parts of sunflowers and the confirmed biological activity of some of them make it possible to assume that green sunflower plants can be regarded, not only as a valuable feed constituent, but also as a source of natural antioxidants. Since the profile of secondary metabolites may change during plant growth [28,29], it seems necessary to consider plants harvested at various growth stages. The aim of this study has therefore been to determine the phenolic compound profile and in vitro antioxidant activity of extracts obtained from the aerial parts of sunflowers harvested at five growth stages, from stem extension to late flowering, to find those that are promising sources of phenolic antioxidants.

## 2. Materials and Methods

### 2.1. Plant Material and Growth Conditions

The study was performed in the Western Po Valley (longitude 7°E, latitude 44°N), Italy. Ornitalia Product Service s.a.s. (Colleredo di Monte Albano, Udine, Italy) provided the sunflower seeds used in the experiment. Plots of 3 × 10 m^2^ were seeded in May and no irrigation or fertilizers were applied during the trial, which ranged from June to July. Sampling was carried out on the basis of a randomized block design after the disappearance of dew and was not performed on rainy days. Three replicates of each sunflower sample were collected (cutting to a 1 to 2 cm stubble height) on subplots of 2 m^2^ at five progressive morphological stages (from stem extension to the late flowering stage). Fresh samples of the whole plants were frozen upon arrival to the laboratory, lyophilized (5Pascal, Trezzano sul Naviglio, Milan, Italy), and then ground to pass through a 1 mm screen.

### 2.2. Chemicals

The reagents 2,2′-azinobis-(3-ethylbenzothiazoline-6-sulfonic acid) (ABTS), butylhydroxyanisole (BHA), β-carotene, chlorogenic acid, 2,2′-diphenyl-1-picrylhydrazyl (DPPH), Folin–Ciocalteau phenol reagent (FCR), formic acid, gallic acid, 6-hydroxy-2,5,7,8-tetramethyl-chroman-2-carboxylic acid (Trolox), linoleic acid, neochlorogenic acid, 2,4,6-tri(2-pyridyl)-s-triazine (TPTZ) and Tween 40 were purchased from Sigma-Aldrich (St. Louis, MO, USA). Acetonitrile, methanol, trifluoroacetic acid and the remaining reagents were obtained from Avantor Performance Materials (Gliwice, Poland).

### 2.3. Extraction

The crude extracts were obtained from lyophilized sunflower samples using 80% (v/v) methanol at 65 °C and at a 1:10 (v/w) material-to-solvent ratio [28]. The extraction was repeated three times. Methanol was removed from connected filtrates by means of evaporation under vacuum (Rotavapor R-200, Büchi Labortechnik, Flawil, Switzerland). The complete drying of the extract was achieved by means of lyophilization (Lyph Lock 6 freeze-dry system, Labconco, Kansas City, MO, USA). The extraction yield was calculated on a matter weight basis.

### 2.4. Determination of the Total Phenolic Content

The assay with the FCR was performed to analyze the total phenolic content (TPC) of sunflower plant extracts and plant fresh matter (FM) [30]. The results were expressed as mg of gallic acid equivalents (GAE) per g of extract or per g of plant FM.

### 2.5. Identification and Quantification of the Phenolic Compounds

Phenolic compounds were detected using an Eksigent microLC 200 system coupled with a TripleTOF 5600+ mass spectrometer (AB Sciex, Framingham, MA, USA). Electrospray ionization was conducted in negative mode and the mass spectrometry (MS) operating conditions were as follows: Ion spray voltage, 4.5 kV; turbo spray temperature, 350 °C; nebulizer gas (GS1) and curtain gas flow rate, 30 L/min; heater gas (GS2) flow rate, 35 L/min; declustering potential (DP) and collision energy (CE) for full-scan MS, 90 V and 20 eV, respectively, and 80 V and 30 eV, respectively, for MS^2^ mode. The time-of-flight (TOF) MS scan was operated at the 100–1200 *m/z* mass range. Chromatographic separation was performed in an Eksigent Halo C18 column (0.5 × 50 mm, 2.7 µm; AB Sciex). The mobile phase, which consisted of 0.1% (v/v) formic acid in water (solvent A) and 0.1% (v/v) formic acid in acetonitrile (solvent B), was pumped into the column in a 1–90% B linear gradient system within 3 min.

An additional separation was performed, to quantify the phenolic compounds, using a Shimadzu HPLC system (Shimadzu, Kyoto, Japan) with an SPD-M10AVp diode-array detector (DAD) and a Luna C18 column (4.6 × 250 mm, 5 µm; Phenomenex, Torrance, CA, USA). The extract solution (10 mg/mL) was passed through a 0.45 µm nylon filter membrane (Sigma-Aldrich, St. Louis, MO, USA) and a portion (20 µL) was injected into the column. The elution was carried out for 30 min in a linear gradient system of 5–26% (v/v) acetonitrile in water acidified with 0.1% (v/v) trifluoroacetic acid, and at a flow rate of 1 mL/min. The DAD was set over a 200 to 400 nm wavelength range. Neochlorogenic and chlorogenic acids were used as standards to express the detected compound content in the sunflower extracts and the FM of the plants.

### 2.6. Determination of the Trolox Equivalent Antioxidant Capacity

The Trolox equivalent antioxidant capacity (TEAC) was assayed using the method of Re et al. [31]. ABTS^•+^ was activated using sodium persulfate according to the original method. Twenty µL aliquots of plant extract solutions (3 mg/mL) were mixed with 2 mL of the ABTS^•+^ solution (stock solution diluted with methanol to an absorbance of 0.720 at 734 nm). The absorbance of the mixtures was recorded at 734 nm (DU-7500 spectrophotometer, Beckman Instruments, Fullerton, CA, USA) after the samples had been incubated at 37 °C for 6 min. The TEAC values were expressed as mmol Trolox equivalents per g of extract or μmol Trolox equivalents per g of plant FM.

### 2.7. Determination of the Ferric-Reducing Antioxidant Power

The ferric-reducing antioxidant power (FRAP) was determined according to a method previously described by Benzie and Strain [32]. A 75 μL aliquot of extract solution in water (1 mg/mL) was vortexed with 2.25 mL of a FRAP reagent and 225 μL of water, and both were warmed to 37 °C. The FRAP reagent was obtained by stirring 10 volumes of acetate buffer solution (300 mM, pH 3.6), 1 volume of 10 mM TPTZ in 40 mM HCl and 1 volume of 20 mM FeCl_3_·6H_2_O. The absorbance was measured at 593 nm (DU-7500 spectrophotometer) after the samples had been incubated at 37 °C for 30 min (TH-24 block heater, Meditherm, Warsaw, Poland). The FRAP values were expressed as mmol Fe^2+^ (FeSO_4_·7H_2_O) equivalent per g extract or μmol Fe^2+^ equivalent per g of plant FM.

### 2.8. Determination of the DPPH Radical Scavenging Activity

The DPPH^•^ scavenging activity of the sunflower extracts was monitored using a method developed by Brand-Williams et al. [33]. A 0.1 mL aliquot of extract solution in methanol, containing between 1.2–6.0 mg of extract, was stirred with 2 mL of methanol and 0.25 mL of DPPH^•^ in methanol (1 mM). The mixture was left to stand at an ambient temperature in the dark for 20 min and absorbance was then measured at 517 nm (DU-7500 spectrophotometer). The concentration of extract in the reaction mixture needed to scavenge 50% of the initial DPPH^•^ (EC_50_) was calculated on the basis of the absorbance curves vs. extract concentration.

### 2.9. β-Carotene–Linoleic Acid Emulsion Oxidation

The ability of sunflower extracts to inhibit β-carotene–linoleic acid emulsion oxidation was determined according to a procedure described by Orak et al. [34], based on Miller’s method [35]. Aliquots of 250 µL of emulsion were oxidized with 20 µL of extract solution (1 mg/mL), BHA solution (0.5 mg/mL) or methanol (control) in a 96-well plate. The mixtures were incubated in an Infinite M1000 microplate reader (Tecan, Männedorf, Switzerland) at 42 °C for 180 min. The absorbance was recorded during incubation at 470 nm at 15 min intervals. The percentage of non-oxidized β-carotene was calculated for each measuring point.

### 2.10. Statistical Analysis

Three sunflower plant samples were collected for each growth stage and extracts were prepared separately for each sample. The extract analyses were performed at least in triplicate. Significant differences among sunflowers at various growth stages were examined by one-way analysis of variance (ANOVA) with Fisher’s least significant difference (LSD) post hoc test. Differences were considered significant at *p* < 0.05. The linear correlations between variables (TPC, sum of phenolics determined by HPLC–DAD and antioxidant activities) were evaluated and Pearson’s correlation coefficients were calculated. The statistical analyses were carried out using GraphPad Prism software (GraphPad Software, San Diego, CA, USA).

## 3. Results and Discussion

The use of 80% (v/v) methanol allowed 26.2% to 32.2% of matter to be extracted from freeze-dried sunflower plants at various growth stages (Table 1). The extracts obtained from most samples had a comparable yield (*p* ≥ 0.05). However, a slightly lower (*p* < 0.05) extraction yield was obtained for the plants at the late flowering stage. The extracts contained phenolic compounds in amounts ranging from 17.6 to 29.3 mg GAE/g, which correspond to 0.54–1.03 mg GAE/g of plant FM (Table 1). The TPC of the extract and plant FM was significantly higher (*p* < 0.05) at the mid- flowering stage than at the other growth stages. The use of polar solvents for the extraction of phenolic compounds causes the main ballast substances of the plant crude extracts to be carbohydrates [36].

Sunflower seed cakes are considered as a potential source of bioactive compounds, due to their high phenolic compound content and antioxidant activity [18]. It was found that the TPC of an aqueous methanol extract of defatted seed meal was 16.1 mg/g [37] and the TPC of the dry weight of a seed cake was evaluated at a level of 247 mg/100 g [18]. The TPC of the aerial parts of sunflowers (Table 1) was 1.1–1.8 and 2.3–3.8 times higher, respectively (the dry weight was taken into account in the latter case). It was also significantly higher than the TPC found for extracts of other sunflower by-products—mature heads with the seeds removed [38]. The TPC of the aforementioned extracts, obtained with a different solid-to-aqueous ethanol ratio, was 4.64–13.55 mg GAE/g. Moreover, the aerial parts of the sunflowers (Table 1) had around a three to five times lower TPC than ray and disk florets [24]. The increased proportion of florets, compared to other parts (leaves, shoots), may explain the increase in the TPC of plants harvested between the early flowering and mid-flowering stages (Table 1). Nevertheless, the TPC of the aerial parts of sunflowers, with respect to those of sunflower by-products, still deserves attention.

**Figure 1:**
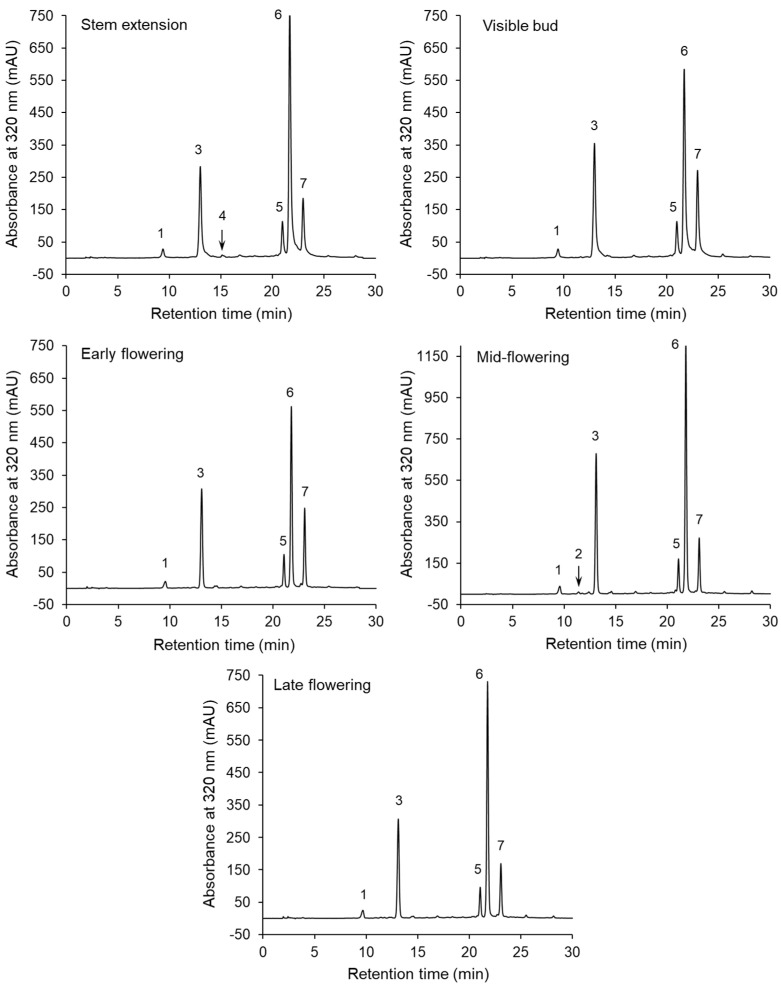
High-performance liquid chromatography with a diode-array detector (HPLC–DAD) separations of the extracts of the aerial parts of sunflowers harvested at various growth stages.

The chromatographic separations of the phenolic compounds of the sunflower extracts are shown in Figure 1. The peaks on the chromatograms correspond to compounds **1–7**, whose UV–DAD spectra are presented in Figure S1 in Supplementary Materials. The HPLC–DAD and LC–MS/MS data and MS/MS spectra used to identify these compounds are shown in Table 2 and Figure S2, respectively. Compounds **1** and **3** were identified as neochlorogenic and chlorogenic acids, respectively, on the basis of a comparison of their retention times and UV spectra with those of commercial standards. Furthermore, both compounds showed an [M–H]^–^ parent ion at *m/z* 353 and MS^2^ base fragment ion at *m/z* 191 (quinic acid moiety). Moreover, a low intensity of the fragment ion at *m/z* 179 (caffeoyl moiety) was noted for compound **3**, but not for compound **1** (Figure S2). This fragmentation pathway was in line with literature findings [21,39]. Neochlorogenic and chlorogenic acids were present in the extracts obtained from the sunflowers harvested at all the growth stages. On the other hand, compound **4** was only detected at the steam extension stage (Figure 1). Compound **4** was identified as cryptochlorogenic acid because, in agreement with literature data [21,39], it exhibited an [M–H]^–^ parent ion at *m/z* 353 and an MS^2^ base fragment ion of [quinic acid–H–H_2_O]^–^ at *m/z* 173. The UV spectrum with λ_max_ at 325 nm and the shoulder at a shorter wavelength, as well as parent and fragment ions at *m/z* 341 and 179 (Table 2), respectively, allowed compound **2** to be tentatively identified as caffeic acid hexose, which was present in one extract (at the mid- flowering stage). Three compounds (**5–7**) showing [M–H]^–^ ions at *m/z* 515, and the MS^2^ base fragment ions at *m/z* 353 were found to be dicaffeoyquinic acid isomers. Further identification of these diacyl-quinic acids was performed, on the basis of the intensity of the MS/MS ions (Figure S2), using the hierarchical scheme proposed by Clifford et al. [39,40]. The result was that compounds **5**, **6** and **7** were identified as 3,4-di-*O*-caffeoylquinic acid, 3,5-di-*O*-caffeoylquinic acid and 4,5-di-*O*-caffeoylquinic acid, respectively. These dicaffeoyquinic acid isomers were contained in the sunflowers at all the growth stages.

The phenolic compounds identified in our study had previously been found in various morphological parts of sunflowers. Fernandez et al. [25] identified three monocaffeoylquinic acids (3-*O*-caffeoylquinic, 4-*O*-caffeoylquinic and 5-*O*-caffeoylquinic acids) and two dicaffeoylquinic acids (3,4-*O*-dicaffeoylquinic and 3,5-*O*-dicaffeoylquinic acids) in sunflower leaves. Masson et al. [26] detected 3-*O*-caffeoylquinic acid, 4-*O*-caffeoylquinic acid and four dicaffeoylquinic acid isomers in leaves. They also found hydroxycinnamaic acid derivatives, including salicylic acid glucoside, syringic acid (formide adduct) and isomers of *p*-coumaroylquinic and feruloylquinic acids. Chlorogenic acid, caffeic acid hexosides and four dicaffeoylquinid acids (3,4-di-*O*-caffeoylquinic, 1,5-di-*O*-caffeoylquinic, 3,5-di-*O*-caffeoylquinic and 4,5-di-*O*-caffeoylquinic acids) have also been determined from extractable phenolic compounds of sunflower ray and disk florets [23]. However, flavonoids, which were previously identified in the leaves and flowers [23,25,26], were not detected in present study in the aerial parts of the sunflowers.

The results of the quantitative analysis of the main phenolic compounds of the extracts and FM of sunflowers at various growth stages are presented in Table 3; Table 4, respectively. The predominant compound throughout the sunflower growth cycle was 3,5-di-*O*-caffeoylquinic acid, and its content ranged from 11.14 to 20.45 mg/g extract, which corresponds to 0.306–0.722 mg/g FM. The chlorogenic acid content was almost two times lower in both the extract and plant FM (6.42–12.30 mg/g extract and 0.175–0.435 mg/g FM, respectively). The other compounds that were observed in the extract and FM, with decreasing content, were as follows: 4,5-di-*O*-caffeoylquinic acid > 3,4-di-*O*-caffeoylquinic acid > neochlorogenic acid. The two compounds (caffeic acid hexose and cryptochlorogenic acid), which were only identified at some growth stages, were found in traces (<0.005 mg/g FM) and are therefore not presented in Table 3 and Table 4. The individual phenolic compounds were more abundant in the extract and FM of plants at the mid-flowering stage, although it should be mentioned that chlorogenic acid and 4,5-di-*O*-caffeoylquinic acid were present at the late flowering and stem extension stages, respectively, with similar (*p* ≥ 0.05) amounts as at the mid-flowering stage. Among the phenolic compounds, the 4,5-di-*O*-caffeoylquinic acid content was the most varied (*p* < 0.05) over the growth stages. Interestingly, the content of this dicaffeoyquinic acid isomer was low in the extract obtained from sunflowers at the late flowering stage, but this was not the case for the other phenolic compounds.

Chlorogenic acid has been determined as the main phenolic compound of sunflower seeds and it constitutes 43–73% of the total phenolic content when extracted from kernels [21,22]. Dicaffeoylquinic acids have been found to be present in smaller amounts and the monocaffeoylquinic acid-to-dicaffeoylquinic acid range is from 5.6:1 to 17.5:1 [19,21]. Our finding indicates an inverse proportion and a predominant content of dicaffeoylquinic acids in the aerial parts of sunflowers at all the growth stages (Table 3 and Table 4). This corresponds to reports of sunflower ray and disk florets in which the diacyl-quinic acid content was higher than that of chlorogenic acid [23,24]. However, it should be mentioned that the 1,5-*O*-dicaffeoylquinic isomer was the main one in the florets, while 3,5-di-*O*-caffeoylquinic acid was the main one in the aerial parts of sunflowers.

The changes in TPC and individual phenolic content during the sunflower growth cycle reported in the present study can be linked with the physiological role of phenolic compounds in plants. In sunflower tissues, caffeoylquinic acids are considered to be precursors of coumarins (e.g., scopoletin, scopolin and ayapin) which, in turn, play a role in biotic and abiotic stress resistance [26,41,42]. Prats et al. [42] reported that the total content of soluble phenolics in sunflower capitula was correlated with resistance to *Sclerotinia sclerotiorum*. In turn, Fernandez et al. [25] selected caffeoylquinic acids as putative markers of drought stress in sunflower leaves. In our study, it seems that the higher accumulation of phenolic compounds during the flowering period (Table 3 and Table 4) could be due to an increased risk of exposure to pathogen infections at this growth stage. On the other hand, caffeoylquinic acids are involved in the lignification of sunflower tissues [43]. The pool of phenolic compounds bound into lignin may increase as plants grow older. Liang et al. [23] found that bound phenolic compounds accounted for about 10% of total phenolics in sunflower florets. It can therefore be assumed that the content of phenolic compounds in plant FM, especially at the later growth states, determined in our study, was probably slightly underestimated by the bound phenolics, which were not extractable with aqueous methanol.

The sum of phenolic compounds in the aerial parts of sunflowers harvested at various growth stages determined by HPLC–DAD correlated closely with the TPC. The correlation coefficients (r) were 0.961 and 0.987 for results expressed on the basis of the extracts and FM, respectively. Moreover, the relationships between TPC and contents of most of the individual phenolic compounds were significant. Very high r values were found for correlations of TPC with 3,4-di-*O*-caffeoylquinic acid and 3,5-di-*O*-caffeoylquinic acid contents (0.977 and 0.968, respectively, for results expressed on the basis of the extracts and 0.989 and 0.991, respectively, for results based on the FM). The correlations between TPC and the neochlorogenic and chlorogenic acid contents were also significant (r of 0.875 and 0.879, respectively, for the extracts and 0.914 and 0.956, respectively, for the FM). Only the relationship between the TPC and the 4,5-di-*O*-caffeoylquinic acid content was not significant (r of 0.699) for the extracts.

The antioxidant activity of sunflower extracts obtained from plants harvested at various stages of the growth cycle was determined as the ability to scavenge free radicals (ABTS^•+^ and DPPH^•^) and to reduce ferric ions. The results of these determinations are presented in Table 5 as TEAC, EC_50_ and FRAP values, respectively. The TEAC of the extracts at the stem extension, visible bud and early flowering stages was comparable (*p* ≥ 0.05) and within the 0.20–0.22 mmol TE/g extract range. No difference in TEAC was observed for the sunflower FM for the first 50 days of growth (6.05–6.16 µmol TE/g FM). In addition, the aforementioned growth stages did not lead to differences in the extract and plant FM, in terms of FRAP. The TEAC of the extract and plant FM increased significantly (*p* < 0.05) between the early flowering and mid-flowering stages, as did the FRAP of the plant FM. The values determined in the next growth stage—late flowering—did not differ significantly (*p* ≥ 0.05) from those at the mid-flowering stage in either assay. The DPPH^•^ scavenging activity of the mid-flowering stage extract was significantly higher (*p* < 0.05) than that at the visible bud stage. The EC_50_ values were 0.13 and 0.21 mg/mL, respectively. The antiradical activity and reduction power of the extracts of the aerial parts of sunflowers, like the previously mentioned TPC, were comparable or higher than those reported for other sunflower by-products [17,18,37]. For example, 0.2 mg of aqueous methanol and the aqueous acetone extracts of defatted seed meal were necessary to reduce the DPPH^•^ by 50% [37]; the TEAC and FRAP of extracts from kernels, hulls and pressed residue ranged from 5.7–89.1 µmol TE/g extract and 12.3–68.6 µmol Fe^2+^/g extract, respectively [17].

The TEAC, FRAP and DPPH^•^ scavenging activity (EC_50_) of the extracts, at various growth stages, were closely correlated with the TPC (r of 0.961, 0.916 and -0.984, respectively) and the sum of phenolic compounds determined by HPLC–DAD (r of 0.901, 0.770 and −0.930, respectively). Significant correlations were also found between the results of the antioxidant activity (TEAC and FRAP) on the basis of the sunflower FM and TPC (r of 0.977 and 0.948, respectively), as well as the sum of phenolic compounds (r of 0.959 and 0.890, respectively). The high r values of these correlations may be due to the similar antioxidant activity of the main phenolic compounds and their comparable contribution to the activity of the extract. In a previous study, Amakura et al. [20] found that the oxygen-radical absorbance capacity of 3,5-di-*O*-caffeoylquinic acids isolated from sunflower seeds was only slightly higher than that of chlorogenic acid, and that the activity of 4-*O*-caffeoylquinic and 3-*O*-caffeoylquinic acids was only about 20% lower. In turn, Kim et al. [44] reported no difference in DPPH^•^ scavenging activity of 3,5-di-*O*-caffeoylquinic and 3,4-di-*O*-caffeoylquinic acids. The abovementioned correlations indicate the main role of phenolic compounds in the antioxidant activity of the extracts of the aerial parts of sunflowers at all growth stages. In the sunflower seeds and leaves, non-phenolic antioxidants have also been determined, including tocopherols and volatile compounds [27,45]. However, their presence in the extracts, in our study, was limited by the extraction conditions: the polar solvent and high temperature of the process. In addition, no condensed tannins were present in the extract, which was found experimentally, based on the vanillin assay (details of the determination are given in Supplementary Materials as Method S1).

High correlation coefficients were obtained when the results of antioxidant assays were correlated. These coefficients amounted to 0.914 (TEAC/FRAP), −0.920 (TEAC/EC_50_) and −0.931 (FRAP/EC_50_) for the extracts and 0.964 (TEAC/FRAP) for the plant FM. Significant correlations between TPC, TEAC, FRAP and EC_50_ were also found in a previous study, by means of principal component analysis, on extracts of the aerial parts of other plants (amaranth and false flax) collected at various growth stages [28,46].

**Figure 2:**
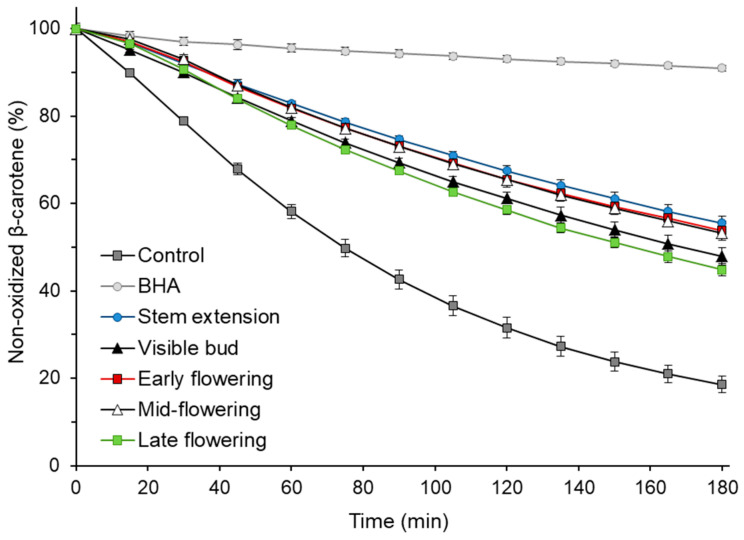
Antioxidant activity of the sunflower plant extracts at various growth stages in the β-carotene–linoleic acid emulsion; BHA, butylhydroxyanisole.

The antioxidant activity of sunflower extracts was also determined at various growth stages using β-carotene–linoleic acid emulsion. The ability of the extract to inhibit the oxidation of the model system is shown in Figure 2. The addition of extracts to the emulsion resulted in 2.4–3.0 times more non-oxidized β-carotene after 180 min of the process compared to a control without an antioxidant. Considering the results of the statistical analysis, the extracts may be divided into two groups, the first, with higher antioxidant activity in the β-carotene–linoleic acid system, consisted of extracts obtained at the stem extension, early flowering and mid-flowering stages, and the second (visible bud and late flowering stages), with less ability to inhibit the oxidation of emulsion. Interestingly, no statistically significant correlation (*p* ≥ 0.05) was found between the percentage of non-oxidized β-carotene after 180 min of oxidation of the emulsion with the addition of extracts and the TPC of those extracts. The correlations of the TEAC, FRAP and DPPH^•^ scavenging activity with the results of the antioxidant activity in the β-carotene–linoleic acid system were not significant either (*p* ≥ 0.05). This phenomenon could be due to the different polarity of the antioxidant activity measurement systems and the different activities of the compounds present in the extracts under polar conditions (ABTS, FRAP and DPPH assays) and in the lipid emulsion system.

## 4. Conclusions

Our study shows, for the first time, the differences in the antioxidant potential and phenolic compound profile of the aerial parts of sunflowers during the growth cycle. The extracts and fresh matter of sunflowers collected from mid-flowering plants were richer in phenolic compounds than those harvested at earlier (stem extension, visible bud and early flowering) and later (late flowering) growth stages. This result was determined considering the total phenolic content assayed with Folin–Ciocalteau phenol reagent, the sum of phenolic compounds obtained by HPLC–DAD and the contents of most individual phenolic compounds. The main phenolic compounds observed throughout the entire sunflower growth cycle were mono- and dicaffeoylquinic acids with the highest content being of 3,5-di-*O*-caffeoylquinic acid. Phenolic compounds were responsible for the antiradical activity and reducing power of the sunflower extracts in the ABTS, DPPH and FRAP assays, as evidenced by significant correlations of the total phenolic content with the results of these antioxidant assays. The contribution of the phenolic compounds to antioxidant activity was not so obvious in the β-carotene–linoleic acid emulsion system. The highest antioxidant activity in the ABTS, DPPH and FRAP assays was generally determined in older plants (mid-flowering and late flowering stages).

Given the above, the aerial parts of sunflowers, in particular those collected at the mid-flowering stage, and their obtained extracts can be considered as a source of phenolic compounds, that is, mono- and dicaffeoylquinic acids, which are characterized by high antioxidant activity, particularly under polar conditions. This source of natural antioxidants has the potential for future applications both as a health-promoting ingredient of functional food and cosmetics and as an additive to food and cosmetic products that increase their shelf-life.

## Figures and Tables

**Table 1:** Total phenolic content of the sunflower extract and fresh matter (FM) of the plants at various growth stages.

Growth Stage	Days after Sowing	Extraction Yield (%)	Total Phenolic Content	
			mg GAE/g Extract	mg GAE/g FM
Stem extension	37	31.5 ± 0.9 ^a^	21.9 ± 2.3 ^b^	0.61 ± 0.07 ^b^
Visible bud	43	32.2 ± 1.7 ^a^	17.6 ± 2.8 ^b^	0.54 ± 0.11 ^b^
Early flowering	50	31.0 ± 1.4 ^a^	20.4 ± 6.1^b^	0.56 ± 0.24 ^b^
Mid-flowering	57	32.2 ± 0.4 ^a^	29.3 ± 1.8 ^a^	1.03 ± 0.07 ^a^
Late flowering	63	26.2 ± 1.8 ^b^	21.7 ± 3.1 ^b^	0.70 ± 0.10 ^b^

GAE, gallic acid equivalents. Means with different letters in the same column are significantly different (*p* < 0.05).

**Table 2:** Absorption maxima (λ_max_) of the HPLC–DAD UV spectra and the MS/MS characteristic ions of the phenolic compounds identified in the sunflower extracts.

Compound No ^1^	λ_max_ (nm)	[M−H]^−^ (*m/z*)	MS^2^ Ions (*m/z*)	Compound
1	297sh, 324	353	191, 179, 135	Neochlorogenic acid
2	286sh, 325	341	179	Caffeic acid hexose
3	299sh, 325	353	191, 179, 135	Chlorogenic acid
4	298sh, 325	353	191, 179, 173, 135	Cryptochlorogenic acid
5	301sh, 324	515	353, 335, 191, 179, 173	3,4-Di-*O*-caffeoylquinic acid
6	301sh, 327	515	353, 191, 179	3,5-Di-*O*-caffeoylquinic acid
7	301sh, 326	515	353, 191, 179, 173	4,5-Di-*O*-caffeoylquinic acid

^1^ The compound number corresponds to the peak number in Figure 1, sh, shoulder. The MS^2^ base fragment ions (*m/z*) are underlined.

**Table 3:** Individual phenolic compound contents in the sunflower extracts (mg/g extract) obtained from plants at various growth stages.

Compound	Stem Extension	Visible Bud	Early Flowering	Mid-Flowering	Late Flowering
Neochlorogenic acid	0.62 ± 0.09 ^b^	0.57 ± 0.13 ^b^	0.60 ± 0.05 ^b^	1.05 ± 0.09 ^a^	0.92 ± 0.05 ^a^
Chlorogenic acid	6.89 ± 0.12 ^b^	7.42 ± 1.22 ^b^	6.42 ± 0.86 ^b^	12.30 ± 0.52 ^a^	7.04 ± 0.58 ^b^
3,4-Di-*O*-caffeoylquinic acid ^1^	2.16 ± 0.46 ^b^	1.58 ± 0.41 ^b^	1.70 ± 0.09 ^b^	2.87 ± 0.12 ^a^	1.96 ± 0.35 ^b^
3,5-Di-*O*-caffeoylquinic acid ^1^	12.70 ± 3.69 ^b^	11.14 ± 2.24 ^b^	11.30 ± 1.37 ^b^	20.45 ± 0.82 ^a^	12.82 ± 1.60 ^b^
4,5-Di-*O*-caffeoylquinic acid ^1^	4.58 ± 0.71 ^a,b^	2.78 ± 0.35 ^c,d^	3.71 ± 0.69 ^b,c^	4.80 ± 0.39 ^a^	2.60 ± 0.51 ^d^
Sum	26.94 ± 4.76 ^b^	23.50 ± 4.27 ^b^	23.73 ±1.52 ^b^	41.47 ± 1.59 ^a^	25.35 ± 2.93 ^b^

^1^ Expressed as chlorogenic acid equivalents. Means with different letters in the same row are significantly different (*p* < 0.05).

**Table 4:** Individual phenolic compound contents in the fresh matter (mg/g FM) of sunflowers at various growth stages.

Compound	Stem Extension	Visible Bud	Early Flowering	Mid-Flowering	Late Flowering
Neochlorogenic acid	0.017 ± 0.003 ^b^	0.017 ± 0.005 ^b^	0.016 ± 0.002 ^b^	0.036 ± 0.004 ^a^	0.031 ± 0.010 ^a^
Chlorogenic acid	0.193 ± 0.008 ^b^	0.228 ± 0.049 ^b^	0.175 ± 0.044 ^b^	0.435 ± 0.032 ^a^	0.234 ± 0.079 ^b^
3,4-Di-*O*-caffeoylquinic acid ^1^	0.061 ± 0.014 ^b^	0.049 ± 0.015 ^b^	0.046 ± 0.007 ^b^	0.101 ± 0.007 ^a^	0.066 ± 0.028 ^b^
3,5-Di-*O*-caffeoylquinic acid ^1^	0.358 ± 0.109 ^b^	0.342 ± 0.087 ^b^	0.306 ± 0.054 ^b^	0.722 ± 0.053 ^a^	0.422 ± 0.134 ^b^
4,5-Di-*O*-caffeoylquinic acid ^1^	0.129 ± 0.023 ^a,b^	0.086 ± 0.015 ^b^	0.101 ± 0.025 ^b^	0.170 ± 0.018 ^a^	0.088 ± 0.041 ^b^
Sum	0.76 ± 0.15 ^b^	0.72 ± 0.17 ^b^	0.64 ± 0.11 ^b^	1.46 ± 0.11 ^a^	0.84 ± 0.29 ^b^

^1^ Expressed as chlorogenic acid equivalents. Means with different letters in the same row are significantly different (*p* < 0.05).

**Table 5:** Trolox equivalent antioxidant capacity (TEAC), ferric-reducing antioxidant power (FRAP) and DPPH^•^ scavenging activity (expressed as EC_50_) of the sunflower extract and fresh matter (FM) of plants at various growth stages.

Growth Stage	TEAC		FRAP		EC_50_ (mg/mL)
	mmol TE/g Extract	µmol TE/g FM	mmol Fe^2+^/g Extract	µmol Fe^2+^/g FM	
Stem extension	0.22 ± 0.02 ^b^	6.16 ± 0.77 ^b^	0.33 ± 0.04 ^a,b^	9.21 ± 1.05 ^b^	0.18 ± 0.03 ^a,b^
Visible bud	0.20 ± 0.03 ^b^	6.15 ± 1.15 ^b^	0.25 ± 0.10 ^b^	7.79 ± 3.43 ^b^	0.21 ± 0.03 ^a^
Early flowering	0.22 ± 0.05 ^b^	6.05 ± 2.09 ^b^	0.33 ± 0.04 ^a,b^	9.10 ± 0.02 ^b^	0.18 ± 0.05 ^a,b^
Mid-flowering	0.28 ± 0.03 ^a^	10.02 ± 1.22 ^a^	0.39 ± 0.04 ^a^	13.90 ± 1.53 ^a^	0.13 ± 0.01 ^b^
Late flowering	0.24 ± 0.03 ^a,b^	7.84 ± 1.13 ^a,b^	0.34 ± 0.08 ^a,b^	11.63 ± 3.24 ^a,b^	0.18 ± 0.01 ^a,b^

TE, Trolox equivalents. Means with different letters in the same column are significantly different (*p* < 0.05).

## Supplementary Materials

The following are available online at https://www.mdpi.com/2076-3921/9/6/535/s1: Method S1: Determination of the condensed tannin content of the extracts of the aerial parts of sunflowers, Figure S1: UV–DAD spectra of phenolic compounds of the extracts of the aerial parts of sunflowers, Figure S2: MS/MS spectra of phenolic compounds identified in the extracts of aerial parts of sunflowers.
