# Supplementary material for: Sunflower (Helianthus annuus L.) Plants at Various Growth Stages Subjected to Extraction—Comparison of the Antioxidant Activity and Phenolic Profile
Source: Antioxidants (Basel). 2020 Jun 19;9(6):535. doi: 10.3390/antiox9060535 (PMC7346105; doi:10.3390/antiox9060535)
Supplement: Supplementary file 1 [file antioxidants-09-00535-s001.zip › antioxidants-826392-supplementary.pdf]

Supplementary Materials

Sunflower (*Helianthus annuus* L.) Plants at Various Growth Stages Subjected to Extraction—Comparison of the Antioxidant Activity and Phenolic Profile

Francesco Gai [1], Magdalena Karamać [2,*], Michał A. Janiak [2], Ryszard Amarowicz [2] and Pier Giorgio Peiretti [1]

[1] Institute of Sciences of Food Production, National Research Council, 10095 Grugliasco, Italy; francesco.gai@ispa.cnr.it (F.G.); piergiorgio.peiretti@ispa.cnr.it (P.G.P.)
[2] Institute of Animal Reproduction and Food Research, Polish Academy of Sciences, Tuwima 10, 10-748 Olsztyn, Poland; m.janiak@pan.olsztyn.pl (M.A.J.); r.amarowicz@pan.olsztyn.pl (R.A.)
* Correspondence: m.karamac@pan.olsztyn.pl; Tel.: +48-895-234-622

Method S1. Determination of the condensed tannin content of the extracts of the aerial parts of sunflowers.

The vanillin assay [1] was used to determine the content of condensed tannins in the extracts of sunflowers at various growth stages. Briefly, the vanillin/HCl reagent was prepared by dissolving 1.5 g vanillin in 250 mL of 4% (v/v) hydrochloric acid in methanol. Aliquots of 5 mL of this reagent were mixed with 1 mL extract solutions (2 mg/mL). The mixtures were left to stand at ambient temperature for 20 min and absorbance was then measured at 500 nm (Beckman DU 7500 spectrophotometer, Beckman Instruments, Fullerton, CA, USA) against a blank containing extract solutions and 4% (v/v) hydrochloric acid in methanol (without vanillin).

Under the above conditions, the pink color of the reaction products did not develop, and the absorbance values were at the level of the limit of detection. The condensed tannins were not determined in the extracts.

1. Price, M.L.; van Scoyoc, S.; Butler, L.G. A critical evaluation of the vanillic reaction as an assay for tannin in sorghum grain. *J. Agric. Food Chem.* **1978**, *26*, 1214–1218. DOI: https://doi.org/10.1021/jf60219a031

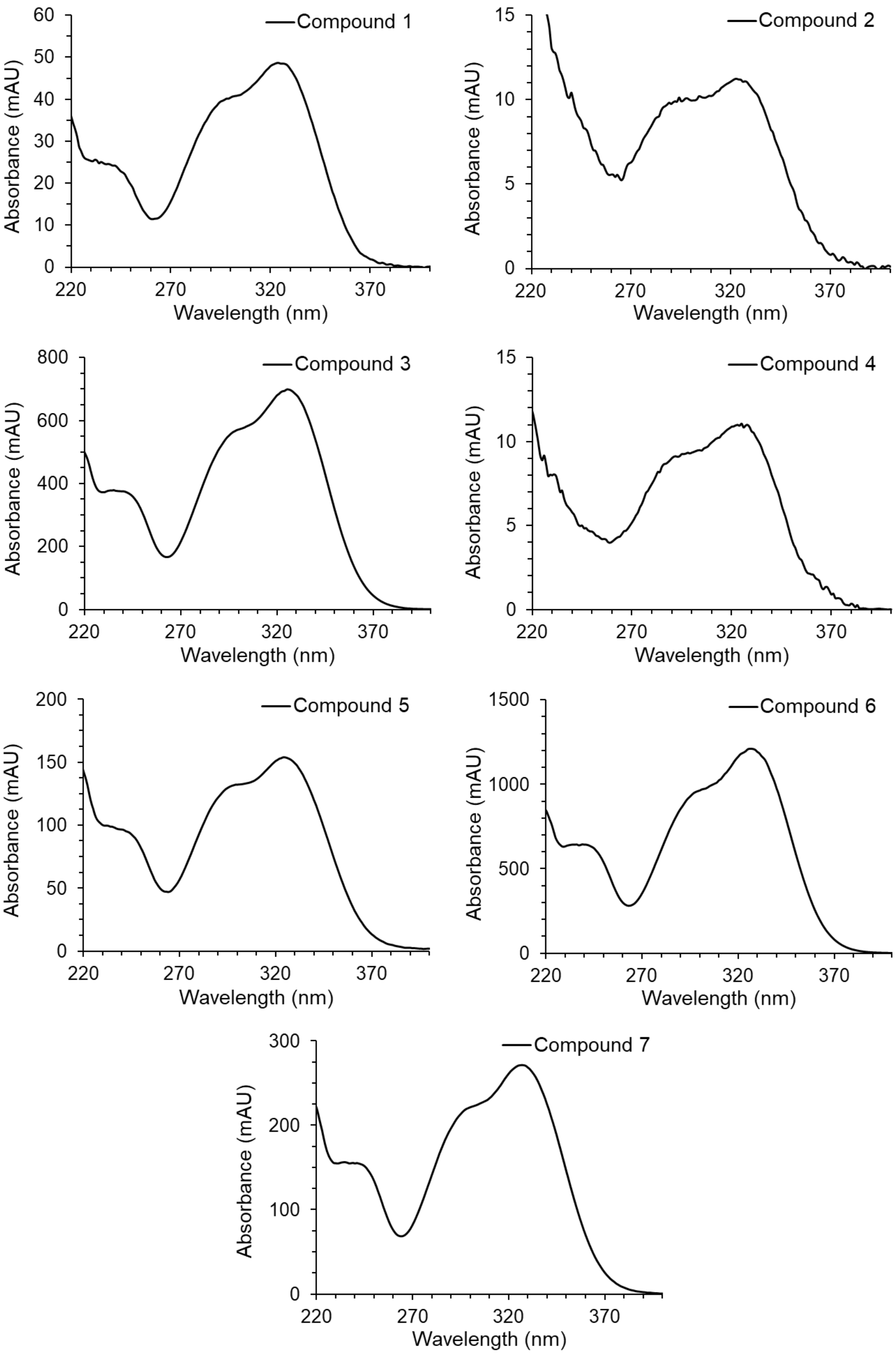

Figure S1. UV-DAD spectra of phenolic compounds of the extracts of the aerial parts of sunflowers. Identified compounds: neochlorogenic acid (**1**), caffeic acid hexose (**2**), chlorogenic acid (**3**), cryptochlorogenic acid (**4**), 3,4-di-*O*-caffeoylquinic acid (**5**), 3,5-di-*O*-caffeoylquinic acid (**6**) and 4,5-di-***O***-caffeoylquinic acid (**7**).

Neochlorogenic acid (compound **1**)

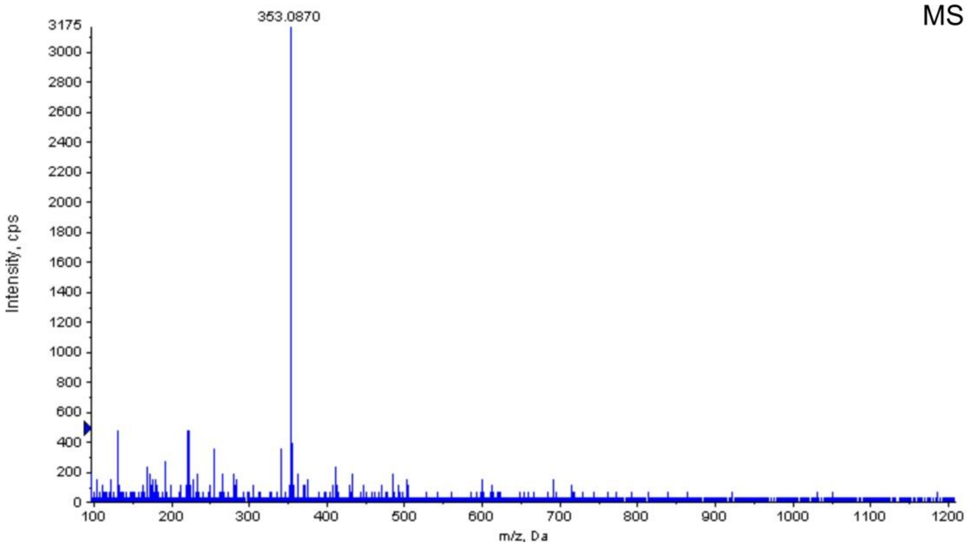

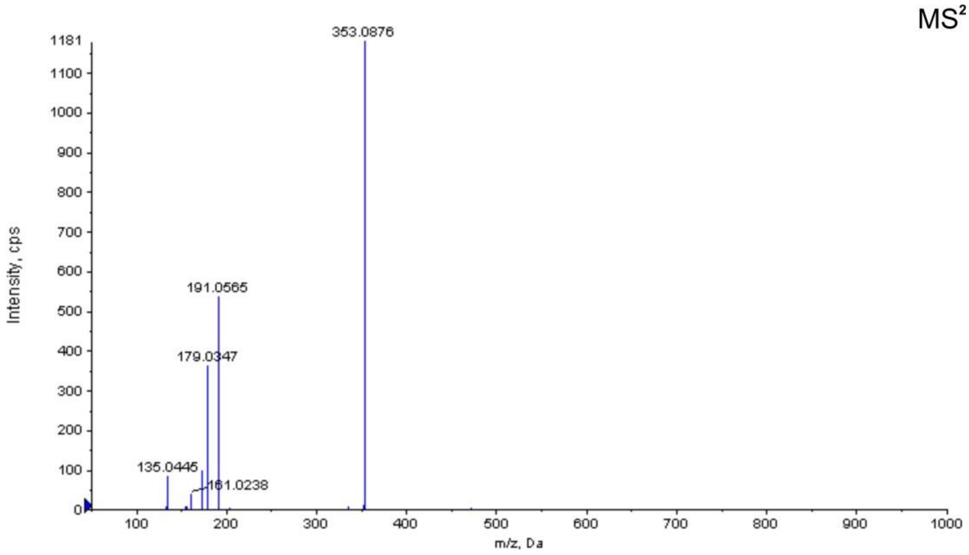

Caffeic acid hexose (compound **2**)

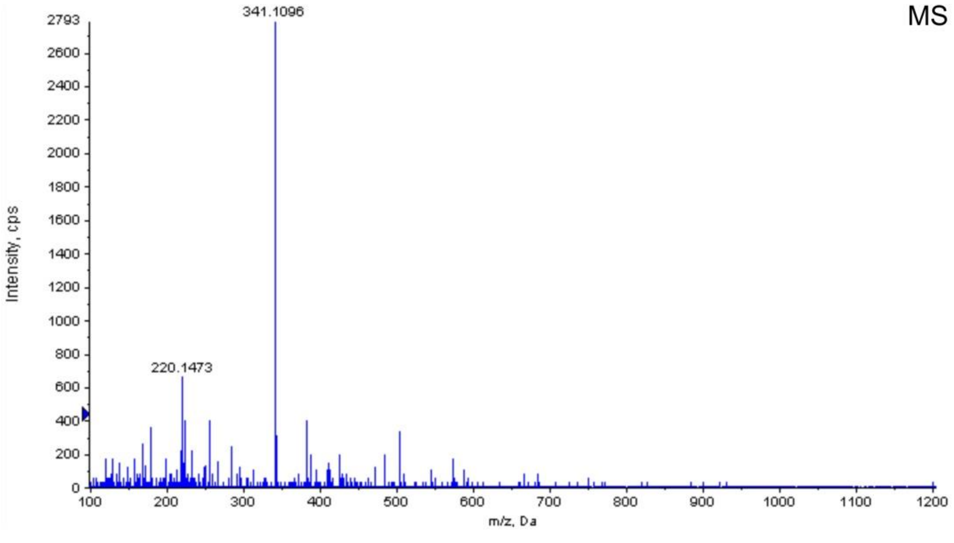

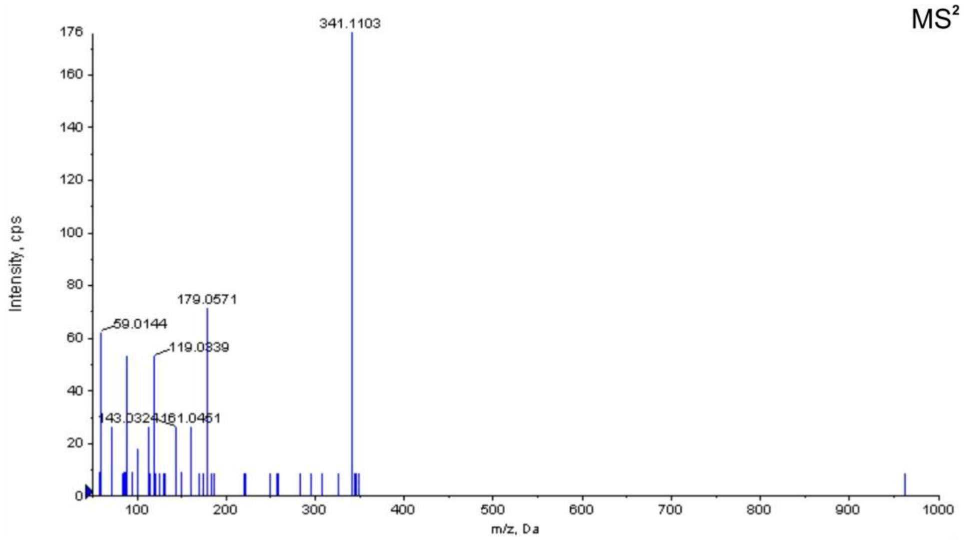

Chlorogenic acid (compound **3**)

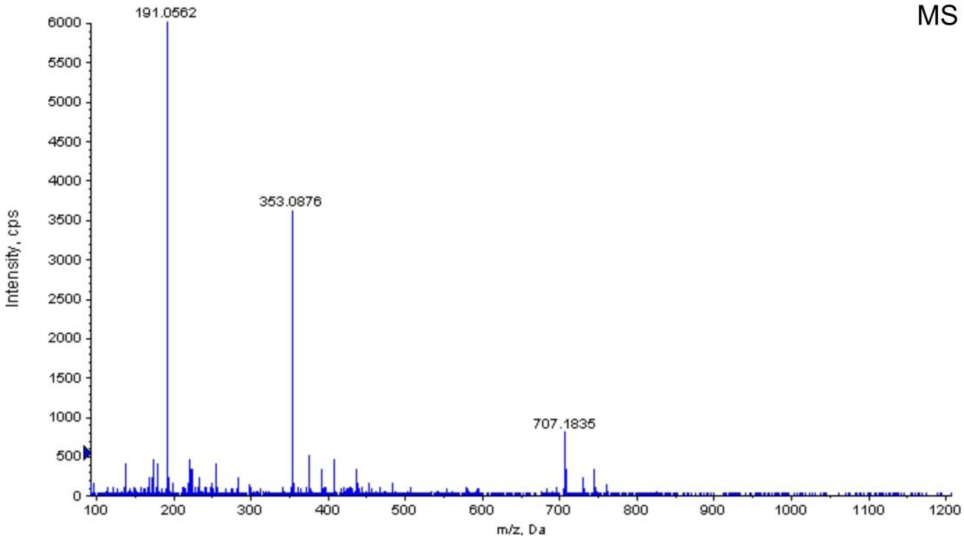

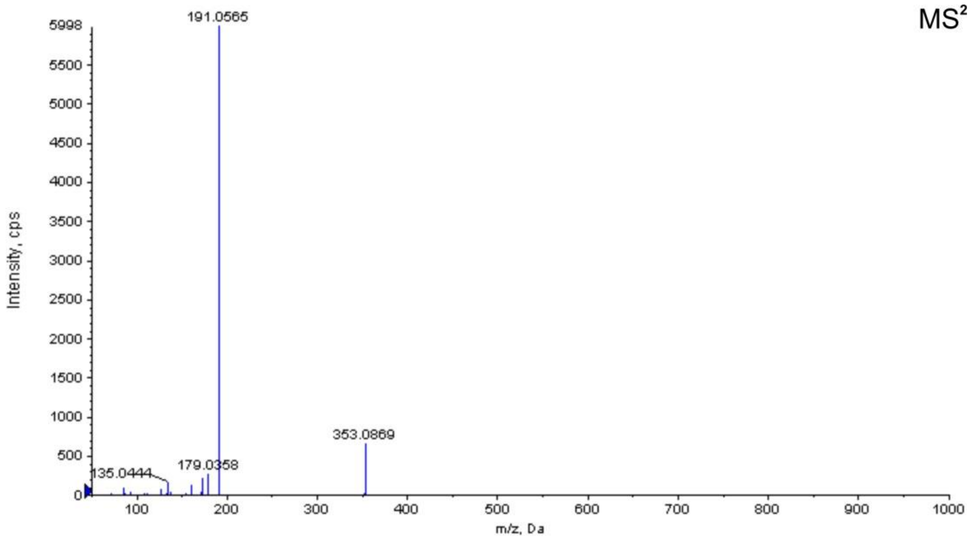

Cryptochlorogenic acid (compound **4**)

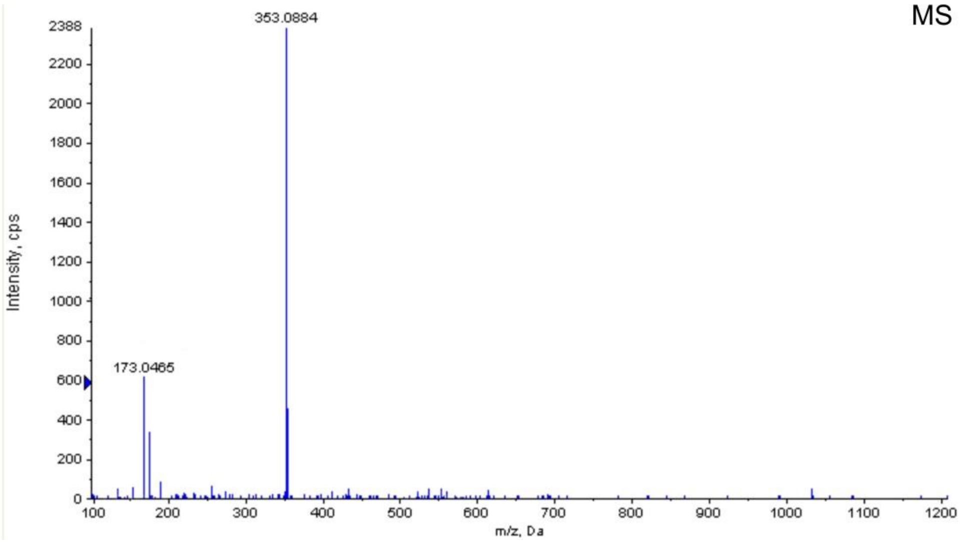

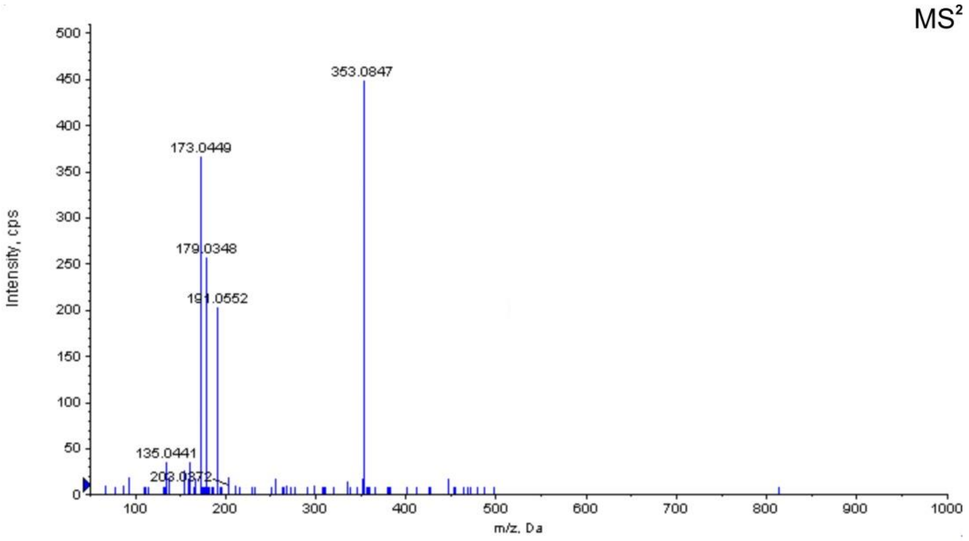

3,4-Di-*O*-caffeoylquinic acid (compound **5**)

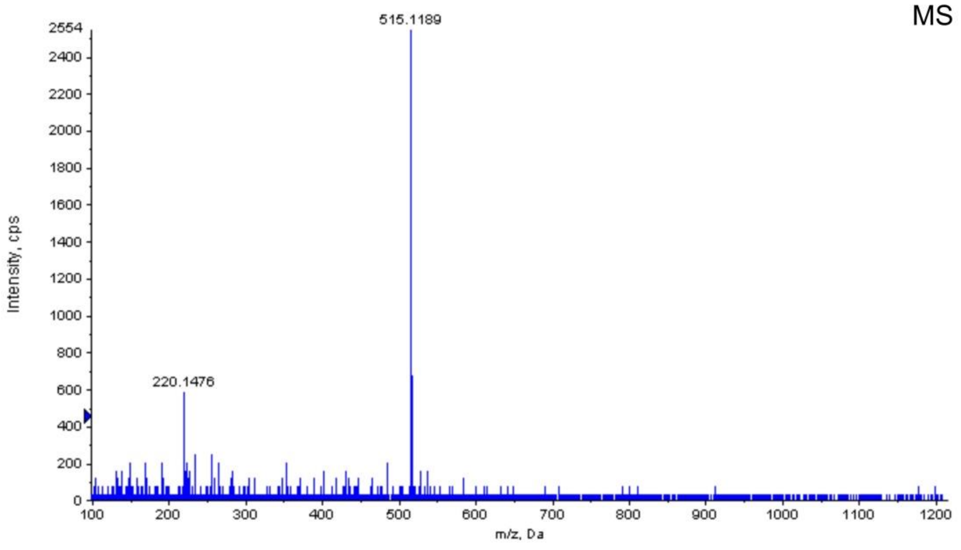

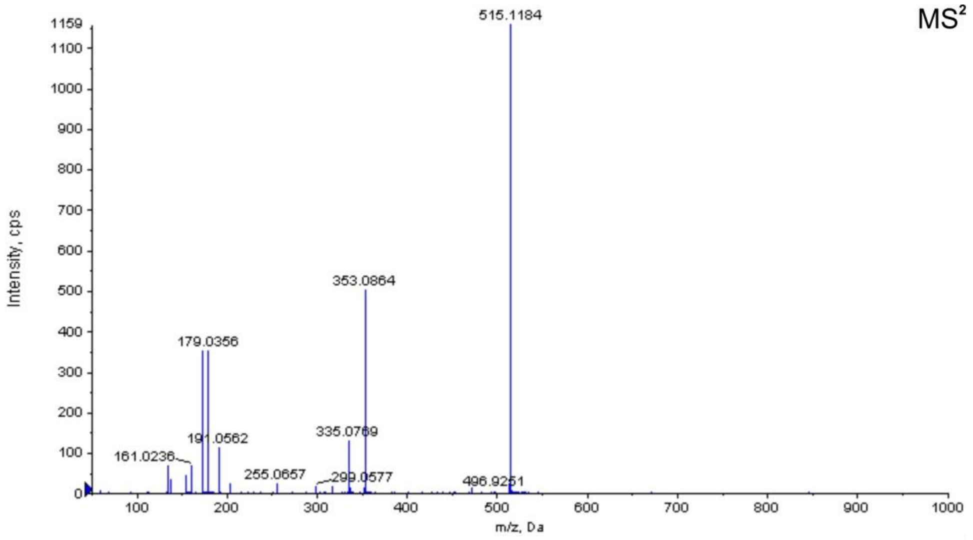

3,5-Di-*O*-caffeoylquinic acid (compound **6**)

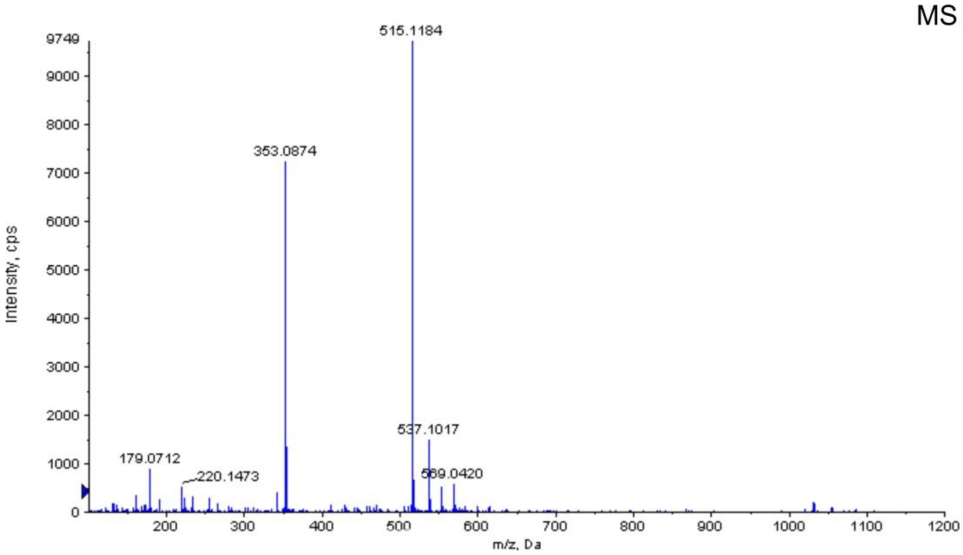

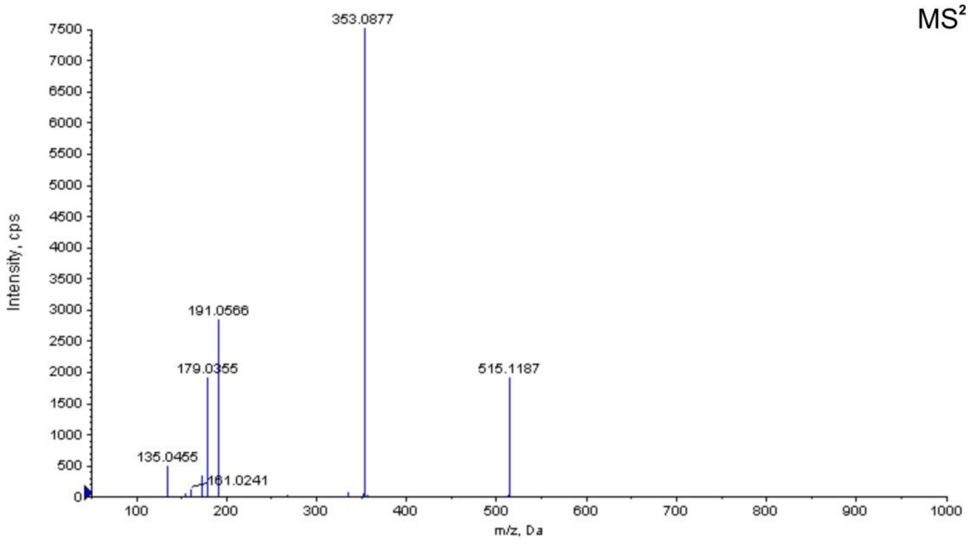

4,5-Di-*O*-caffeoylquinic acid (compound **7**)

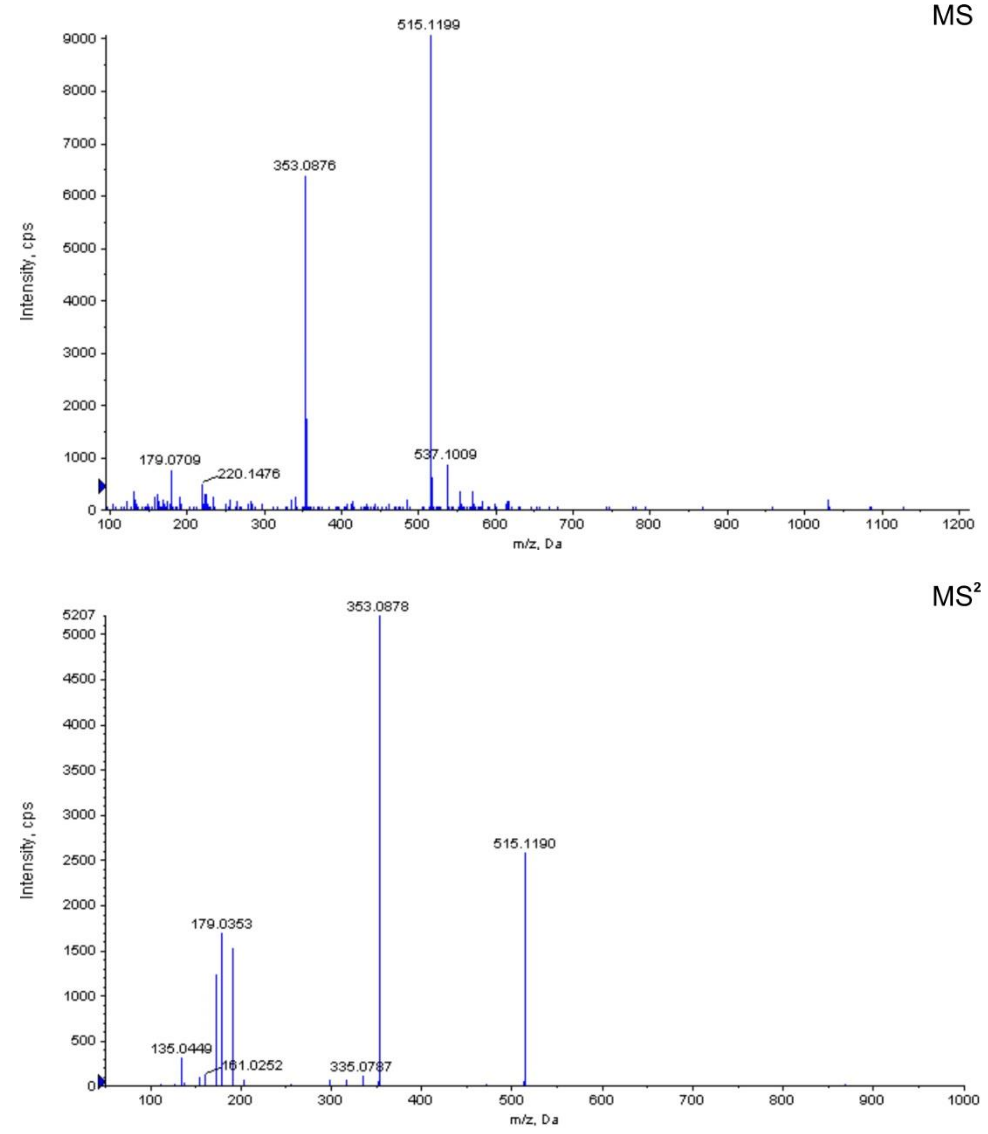

Figure S2. MS/MS spectra of phenolic compounds identified in the extracts of aerial parts of sunflowers.
